# Diabetes-Related Complications and Pancreatic Cancer Incidence in the Multiethnic Cohort

**DOI:** 10.1093/jncics/pkaa035

**Published:** 2020-05-04

**Authors:** Albert J Farias, Anna H Wu, Jacqueline Porcel, Loïc Le Marchand, Lynne R Wilkens, Kristine R Monroe, Gertraud Maskarinec, Stephen J Pandol, Veronica Wendy Setiawan

**Affiliations:** p1 Department of Preventive Medicine, Keck School of Medicine, University of Southern California, Los Angeles, CA, USA; p2 Norris Comprehensive Cancer Center, Los Angeles, CA, USA; p3 Epidemiology Program, University of Hawaii Cancer Center, Honolulu, HI, USA; p4 Division of Gastroenterology, Departments of Medicine, Cedars-Sinai Medical Center, Los Angeles, CA, USA

## Abstract

**Background:**

People with diabetes are at an increased risk of developing pancreatic cancer. However, it is unclear whether diabetes-related complications are associated with risk of pancreatic cancer.

**Methods:**

A nested matched case-control analysis was conducted among the fee-for-service Medicare participants of the prospective Multiethnic Cohort (n = ∼123 000). Between 2001 and 2014, 433 incident cases of pancreatic ductal adenocarcinoma were matched to 1728 controls by birth year, sex, race and ethnicity, and age at cohort entry. Participants were linked to data from the California and Hawaii cancer registries and Medicare claims. We used the diabetes complications severity index (DCSI) for the presence of 7 complications within 2 years prior to the diagnosis date of the index case. Multivariable conditional logistic regression was used to examine the association of DCSI with pancreatic cancer incidence.

**Results:**

Diabetes was present among 45.4% of cases and 34.1% of controls. Cases had higher DCSI score compared with controls (score ≥4: 32.8% in cases; 21.2% in controls). The most prevalent diabetes-related complications for cases were cardiovascular disease (61.2%), nephropathy (31.2%), and cerebrovascular disease (21.7%). Individuals with diabetes (odds ratio [OR] = 1.48, 95% confidence interval [CI] = 1.14 to 1.91), nephropathy (OR = 1.75, 95% CI = 1.32 to 2.33), cardiovascular disease (OR = 1.88, 95% CI = 1.45 to 2.44), and metabolic complications (OR = 6.61, 95% CI = 2.49 to 17.50) were at increased risk of pancreatic cancer. For every 1-unit increase in DCSI score, participants had 18% greater risk of pancreatic cancer (OR = 1.18, 95% CI = 1.11 to 1.25).

**Conclusions:**

Participants with diabetes-related complications have an elevated risk of pancreatic cancer. Identifying diabetes-related complications may help identify high-risk groups who can be studied for development of early markers for this fatal cancer.

Pancreatic cancer is one of the most fatal cancers with an estimated 56 000 new cases and 45 000 deaths associated with the disease in 2019 (1). According to the National Cancer Institute’s Surveillance, Epidemiology, and End Results registry, the 5-year survival rate for pancreatic cancer is 9.3% in 2019. By 2030, pancreatic cancer is predicted to be the second most fatal cancer after lung cancer (2). Because there are no screening tests to detect pancreatic cancer and patients experience symptoms only with advanced-stage disease, there is a critical need to identify epidemiological risk factors and groups at the highest risk of developing the disease. Known risk factors for pancreatic cancer include smoking (3-5), family history of pancreatic cancer (3,5), and obesity (3,5-7).

Diabetes is known to increase the risk of pancreatic cancer (5,8-11), especially among patients diagnosed after the age of 50 years (12). However, most of these studies measure only the presence or absence of diabetes without taking into consideration the severity or heterogeneity of the condition. Uncontrolled and unmanaged diabetes often results in complications such as retinopathy, nephropathy, neuropathy, cerebrovascular, cardiovascular, peripheral vascular, and metabolic disease (13-16). Diabetes complications have been linked to higher health-care costs, utilization, and mortality compared with diabetes with no complications (14,16) but, to our knowledge, have not been studied in relation to pancreatic cancer.

African Americans (17), Latinos (17), and Japanese Americans (18) are at a high risk of developing diabetes and are more likely to have a higher prevalence of severe diabetes complications compared with non-Hispanic whites (15). The reason for severe diabetes among minorities may be due to an underuse of primary care physicians, poor medication adherence, and poor understanding of their disease (19–21). Although African Americans have the highest incidence of pancreatic cancer in the United States (22) and have the highest prevalence of diabetes, the comparably high rate in Latinos is inconsistent with their low rate of pancreatic cancer. This suggests that measuring the presence or absence may not entirely explain the true risk of pancreatic cancer because of glucose abnormalities, and identifying complications from diabetes may help identify high-risk groups.

Understanding the role of diabetes in the etiology of pancreatic cancer can be helpful in targeting efforts to identify populations at high risk such as racial and ethnic minorities who have a higher prevalence of diabetes compared with non-Hispanic whites in the population. However, the relationship between diabetes-related complications and pancreatic cancer is unknown. Therefore, the goal of this study was to investigate whether elevated risk of pancreatic cancer is observed in patients with diabetes-related complications.

## Methods

### Study Population

The Multiethnic Cohort (MEC) is an ongoing population-based prospective cohort study of more than 215 000 participants from Hawaii and California, assembled between 1993 and 1996. The MEC was established to study cancer etiology by collecting detailed information on diet and other lifestyle factors and biospecimens to study genetic factors. The study design and baseline characteristics have been published elsewhere (23). Briefly, the cohort comprises predominately 5 racial and ethnic groups: African Americans, Native Hawaiians, Japanese Americans, Latinos, and whites (aged 45-75 years at cohort entry). All participants completed a self-administered baseline questionnaire on demographic and lifestyle factors, physical activity, and tobacco smoking history.

The MEC was linked with the Centers for Medicaid and Medicare Services claims data to obtain all inpatient and outpatient records for cohort members who were enrolled in Medicare fee-for-service (FFS; n = ∼123 000) (24). The claims data contain dates of services, International Classification of Disease (ICD-9-CM) diagnosis codes, and Concurrent Procedural Terminology (version 4) codes for all billed claims. Medicare claims data between 1999 and 2016 are available in the MEC. Study protocols were approved by the Committee on Human Studies at the University of Hawaii and by the Institutional Review Board of the Keck School of Medicine of University of Southern California.

Eligible participants for the present analysis were FFS cohort members who were free of cancer at cohort entry and had complete risk factor data including smoking status, body mass index (BMI), alcohol intake, and physical activity.

### Case Ascertainment and Matched Control Selection

We used a nested case-control design for this study. Cases were all incident pancreatic cancer cases (diagnosed at aged 67 years or older) identified among the at-risk multiethnic cohort participants with 24 months of Medicare FFS enrollment prior to their cancer diagnosis. First incident primary invasive pancreatic cancer cases (ICD-oncology [ICD-O] version 3 topographic [C250-C259]) were identified from the annual linkage of cohort participants to the California and Hawaii tumor registries. These cancer registries are part of the Surveillance, Epidemiology, and End Results program. To assure at least 24 months between exposure and cancer diagnosis, cases were required to be diagnosed at aged 67 years or older. Cases were diagnosed with primary pancreatic cancer occurring between 2001 and 2014 and enrolled in Medicare FFS for at least 2 consecutive years prior to their diagnosis. We restricted the analysis to pancreatic ductal adenocarcinoma (PDAC), an exocrine tumor that accounts for more than 90% of all pancreatic cancers using histological type of adenocarcinoma and infiltrating pancreatic adenocarcinoma (ICD-O-3 histology codes excluding 9050-9055, 9140, or 9590-9992). A total of 433 incident cases of PDAC were included in this analysis.

Control subjects were members from the same cohort, aged 65 years and older, without a previous history of any cancer with 24 months of follow-up during the same time period as the matched case. Four controls were individually matched to each case on race and ethnicity, age at cohort entry (±1 year), sex, and birth year (±1 year). All controls were cancer-free at the time of selection into the study and were enrolled in Medicare FFS for 24 months prior to the date of diagnosis of the matched case. Between 2001 and 2014, of the incident cases of PDAC, 433 were matched to 1728 controls by birth year, sex, race and ethnicity, and age at cohort entry.

### Diabetes Complications Severity Index (DCSI)

We used the DCSI developed by Young and colleagues (14) to assess the level of risk of adverse outcomes, including hospitalizations and mortality, by using diagnosed complications. A DCSI score ranges from 0 to 13 based on the presence and severity of 7 categories of complications including cardiovascular disease, nephropathy, retinopathy, peripheral vascular disease, stroke, neuropathy, and metabolic complications. Metabolic complications included a diagnosis of ketoacidosis, hyperosmolar, and other coma. Each complication category is scored on a scale from 0 to 2, determined by the presence and severity of the complications (0 = not present, 1 = some abnormality, and 2 = severe abnormality), except for neuropathy (scored 0-1) (14). Diabetes and the presence or absence of each diabetes-related complication (yes or no) were identified based on ICD-9-CM codes from inpatient and outpatient Medicare claims data during the 2-year period prior to a pancreatic cancer diagnosis for cases and during the same calendar period for each matched control (14). We identified DCSI among the entire cohort.

### Covariates

Data on demographic and lifestyle factors including age at cohort entry (years), sex (male or female), education level (less than or equivalent to a high school diploma, some college, or college or higher), neighborhood-level socioeconomic status at the census tract level for the address on file at baseline (quintiles lowest to highest), BMI (kg/m^2^), smoking status (never, former, current), any vigorous physical activity (yes or no), and alcohol intake (g/day) were obtained from the baseline questionnaire. We assessed nondiabetes-related comorbid conditions using Medicare claims data for the 2-year period for congestive heart failure (yes or no), chronic obstructive pulmonary disease (COPD; yes or no), dementia (yes or no), paralysis (yes or no), mild liver disease (yes or no), moderate to severe liver disease (yes or no), peptic ulcer disease (yes or no), and rheumatologic disease (yes or no).

### Statistical Analysis

Frequencies and means were used to compare characteristics between pancreatic cancer cases and controls. Two-sided χ^2^ tests were used to calculate *P* values to measure the association between each covariate and race and ethnicity or DCSI score. Conditional multivariate logistic regression was used to estimate the association of pancreatic cancer with a diagnosis of diabetes and each of the complications. The association between diabetes complications, DCSI, and pancreatic cancer incidence was examined using multivariable conditional logistic regression analyses stratified by matched set and adjusted for baseline educational attainment, neighborhood-level socioeconomic status, alcohol intake, smoking status, any vigorous physical activity at baseline, BMI, and other nondiabetes-related comorbid conditions listed previously. All analyses were performed using SAS 9.2 software (SAS Institute Inc, Cary, NC). Statistical significance was set at *P* less than .05.

## Results

In this nested multiethnic case-control study, the largest racial and ethnic group was Japanese Americans (43.5%) and majority were female (52.6%) (Table 1). The majority of study participants had attended some college or higher (54.0% among cases and 53.9% among controls), were younger than 70 years at cohort entry (76.7% among cases and 78.1% of controls), consumed less than 24 g/day of alcohol (88.5% among cases and 89.5% among controls), and were former or current smokers (54.3% among cases and 52.3% among controls).

**Table 1.** Descriptive characteristics of cases and controls

	Cases (n = 433)	Controls (n = 1728)
Characteristics	No. (%)	No. (%)
Age at cohort entry, y^a^		
<60	116 (26.8)	463 (26.8)
60-64	89 (20.6)	360 (20.8)
65-69	127 (29.3)	526 (30.4)
70-74	89 (20.6)	342 (19.8)
≥75	12 (2.8)	37 (2.1)
Sex^a^		
Male	205 (47.3)	820 (47.5)
Female	228 (52.7)	908 (52.6)
Race/ethnicity^a^		
African American	62 (14.3)	248 (14.4)
Native Hawaiian	40 (9.2)	156 (9.0)
Japanese American	188 (43.4)	752 (43.5)
Latino	60 (13.9)	240 (13.9)
White	83 (19.2)	332 (19.2)
Educational attainment		
High school or less	199 (46.0)	796 (46.1)
Vocational/Some college	128 (29.6)	471 (27.3)
College or higher	106 (24.4)	461 (26.7)
Neighborhood SES, quintiles		
1 (low)	82 (18.9)	292 (16.9)
2	64 (14.8)	210 (12.2)
3	76 (17.6)	306 (17.7)
4	82 (18.9)	259 (15.0)
5 (high)	115 (26.6)	516 (29.9)
Missing	14 (3.2)	145 (8.4)
Health behaviors		
Alcohol intake (g/day)		
<24	383 (88.5)	1547 (89.5)
≥24	50 (11.6)	181 (10.5)
Smoking status		
Never	198 (45.7)	824 (47.7)
Former	167 (38.6)	704 (40.7)
Current	68 (15.7)	200 (11.6)
Any vigorous physical activity (yes)	89 (20.6)	386 (22.3)
Clinical factors and comorbid conditions		
Body mass index (kg/m^2^)		
<25	181 (41.8)	796 (46.1)
25-29.9	181 (41.8)	679 (39.3)
≥30	71 (16.4)	253 (14.6)
Congestive heart failure	57 (13.2)	182 (10.5)
Chronic obstructive pulmonary disease	90 (20.8)	218 (12.6)
Dementia	24 (5.5)	124 (7.2)
Paralysis (hemiplegia or paraplegia)	11 (2.5)	23 (1.3)
Mild liver disease	7 (1.6)	19 (1.1)
Moderate/severe liver disease	6 (1.4)	7 (0.4)
Peptic ulcer disease	15 (3.5)	33 (1.9)
Rheumatologic disease	22 (5.1)	56 (3.2)
Cancer prognostic factors		
Pancreatic cancer stage at diagnosis		
Localized	40 (9.2)	—
Regional	131 (30.3)	—
Metastatic	196 (45.3)	—
Unknown/not specified	66 (15.2)	—
Age at diagnosis, y		
67-69	38 (8.8)	—
70-74	90 (20.8)	—
75-79	112 (25.9)	—
80-84	111 (25.6)	—
≥85	82 (18.9)	—

^a^ Matching variables. Data from baseline questionnaire (age, sex, race/ethnicity, education, health behaviors, body mass index), Medicare claims (diabetes and related complications, comorbid conditions), and Surveillance, Epidemiology, and End Results (cancer prognostic factors). SES = socioeconomic status.

Of the 433 PDAC cases, 21.9% had no diabetes-related complications, 13.6% had 1 complication, 19.2% had 2 complications, 12.4% had 3 complications, 12.0% had 4, and 20.8% had 5 or more complications (Table 2). Among cases, age, sex, race and ethnicity, smoking status, BMI, COPD, dementia, and rheumatologic disease were statistically significantly associated with DCSI (*P* < .05). Among controls, age, sex, race and ethnicity, educational attainment, BMI, COPD, dementia, and rheumatologic disease were statistically significantly associated with the DCSI score (*P* < .05).

**Table 2.** Baseline characteristics by diabetes complications severity index

Characteristics	Diabetes complications severity index							Diabetes complications severity index											
	Cases							Controls											
	0	1	2	3	4	5+	*P* ^a^	0	1		2		3			4		5+	*P* ^a^
	(n = 95)	(n = 59)	(n = 83)	(n = 54)	(n = 52)	(n = 90)		(n = 669)	(n = 236)		(n = 289)		(n = 167)			(n = 172)		(n = 195)	
	%	%	%	%	%	%		%	%		%		%			%		%	
Index age, y							.06												.001
67–69	16.8	10.2	8.4	5.6	3.9	4.4		10.2	8.9		9.3		4.8			5.2		4.1	
70–74	16.8	18.6	28.9	24.1	28.9	13.3		24.5	22.0		19.0		20.4			10.5		14.9	
75–79	24.2	22.0	22.9	20.4	30.8	26.7		24.1	29.2		23.9		27.0			20.9		25.6	
80–84	24.2	28.8	24.1	25.9	23.1	30.0		22.3	25.4		25.6		27.0			33.7		29.2	
≥85	17.9	20.3	15.7	24.1	13.5	25.6		19.0	14.1		22.2		21.0			29.7		26.2	
Sex							.04												.25
Male	39.0	47.5	43.4	50.0	67.3	46.7		46.3	43.6		49.1		43.7			51.2		53.3	
Female	61.1	52.5	56.6	50.0	32.7	53.3		53.7	56.4		50.9		56.3			48.8		46.7	
Race/ethnicity							.001												.001
African American	9.5	11.9	10.8	16.7	11.5	24.2		13.0	7.2		11.8		18.0			13.4		29.2	
Native Hawaiian	8.4	6.8	6.0	7.4	9.6	15.6		9.0	8.5		7.6		8.4			14.0		8.2	
Japanese American	52.6	54.2	48.2	44.4	51.9	16.7		46.6	50.9		48.1		46.7			35.5		21.5	
Latino	4.2	15.3	10.8	18.5	17.3	21.1		11.8	14.4		11.8		9.0			18.0		24.1	
White	25.3	11.9	24.1	13.0	9.6	22.2		19.6	19.1		20.8		18.0			19.2		16.9	
Educational							.40												.03
≤High school	44.2	47.5	39.8	38.9	50.0	54.4		42.3	40.7		49.8		52.7			52.3		48.7	
Some college	28.4	23.7	31.3	33.3	34.6	27.8		28.7	32.6		23.2		19.2			26.2		29.7	
College or higher	27.4	27.1	28.9	27.8	15.4	17.8		28.7	25.9		27.0		27.5			20.4		21.0	
Missing	0	1.7	0	0	0	0		0.3	0.9		0		0.6			1.2		0.5	
Neighborhood SES, quintiles							.67												.001
1 (low)	17.9	20.3	13.3	18.5	13.5	27.8		14.4	14.4		15.9		14.4			22.1		27.7	
2	16.8	6.8	19.3	16.7	17.3	11.1		10.8	12.7		11.1		11.4			16.3		14.9	
3	14.7	17.0	13.3	22.2	25.0	17.8		17.8	13.1		17.3		25.2			19.2		15.9	
4	14.7	23.7	25.3	14.8	17.3	17.8		16.4	16.1		18.0		13.2			7.6		12.3	
5 (high)	31.6	28.8	26.5	24.1	23.1	23.3		32.9	33.9		29.4		28.7			26.7		19.0	
Missing	4.2	3.4	2.4	3.7	3.9	2.2		7.8	9.8		8.3		7.2			8.1		10.3	
Health behaviors																			
Alcohol intake (g/day)							.71												.33
<24	91.6	86.4	91.6	85.2	86.5	86.7		90.0	92.0		87.2		91.6			89.5		86.7	
≥24	8.4	13.6	8.4	14.8	13.5	13.3		10.0	8.1		12.8		8.4			10.5		13.3	
Smoking status							.04												.06
Never	46.3	44.1	47.0	51.9	38.5	45.6		49.0	53.4		47.4		50.9			41.9		39.0	
Former	42.1	40.7	45.8	20.4	44.2	34.4		38.1	37.3		41.2		41.9			47.1		46.7	
Current	11.6	15.3	7.2	27.8	17.1	20.0		12.9	9.3		11.4		7.2			11.1		14.4	
Any vigorous physical activity							.26												.32
No	72.6	88.1	81.9	79.6	75.0	81.1		78.2	74.6		78.5		73.6			76.7		82.6	
Yes	27.4	11.9	18.1	20.4	25.0	18.9		21.8	25.4		21.5		26.4			23.3		17.4	
Clinical factors and comorbid conditions																			
BMI (kg/m^2^)							.03												.001
<25	54.7	45.8	38.6	46.3	25.0	35.6		51.0		48.7		48.1		46.7	37.2		30.3		
25–29.9	35.8	42.4	44.6	38.9	53.9	40.0		38.4		39.0		38.4		34.1	44.2		44.1		
≥30	9.5	11.9	16.9	14.8	21.2	24.4		10.6		12.3		13.5		19.2	18.6		25.6		
COPD							.01												.001
No	87.4	81.4	84.3	81.5	71.1	67.8		92.7		89.4		87.9		86.8	81.4		71.8		
Yes	12.6	18.6	15.7	18.5	28.9	32.2		7.3		10.6		12.1		13.2	18.6		28.2		
Dementia							.03												.001
No	97.9	98.3	95.2	92.6	96.1	87.8		96.1		95.7		91.7		91.0	90.1		83.6		
Yes	2.1	1.7	4.8	7.4	3.9	12.2		3.9		4.3		8.3		9.0	9.9		16.4		
Rheumatologic disease							.02												.001
No	95.8	96.6	95.2	100	98.2	87.8		98.8		96.6		97.2		95.8	94.8		91.8		
Yes	4.2	3.4	4.8	0	1.9	12.2		1.2		3.4		2.8		4.2	5.2		8.2		

^a^ Two-sided χ^2^ test. BMI = body mass index; COPD = chronic obstructive pulmonary disease; SES = socioeconomic status.

In this study, diabetes was present among 45.4% of cases and 34.1% of controls. Compared with controls, cases had a DCSI of 4 or more (32.8% vs 21.2%) and a greater proportion was diagnosed with each of the 7 complications and a greater proportion of controls had no complications (Table 3). The most prevalent diabetes-related complications for cases were cardiovascular disease (61.2%), nephropathy (31.2%), and cerebrovascular disease (21.7%). A greater proportion of PDAC cases compared with controls was diagnosed with neuropathy (19.2% vs 13.4%) and peripheral vascular disease (18.5% vs 12.0%). A greater proportion of Latino cases was diagnosed with diabetes (58.3%) followed by African Americans (50.0%) compared with other racial and ethnic groups. Among African American, Native Hawaiian, and Latino cases, about one-third (35.5%, 35.0%, and 31.7%, respectively) had a DCSI score of 5 or more, a greater proportion than any of the other racial and ethnic groups of cases. Among controls, African Americans and Latinos displayed the greatest proportion of a DCSI score of 5 or more (23.0% and 19.6%, respectively) compared with the other racial and ethnic groups. In contrast, among cases, whites had the highest proportion with a DCSI score of 0 (28.9%) followed by Japanese Americans (26.6%). The most common complication among the cases and controls was cardiovascular disease, ranging from 74.2% in African American cases to 38.0% in Japanese American controls. Nephropathy and cerebrovascular disease were also highly prevalent among cases.

**Table 3.** Descriptive characteristics of diabetes and diabetes-related complications, by race/ethnicity

Chronic condition	Cases							Controls						
	Total	African American	Native Hawaiian	Japanese American	Latino	White	*P* ^a^	Total	African American	Native Hawaiian	Japanese American	Latino	White	*P* ^a^
	%	%	%	%	%	%		%	%	%	%	%	%		
	(n = 433)	(n = 62)	(n = 40)	(n = 188)	(n = 60)	(n = 83)		(n = 1728)	(n = 248)	(n = 156)	(n = 752)	(n = 240)	(n = 332)	
Diabetes	45.5	50.0	45.0	45.7	58.3	32.5	.001	31.4	43.7	39.1	32.9	37.1	17.8	.001
Diabetes-related complications														
Retinopathy	16.4	16.1	22.5	14.4	23.3	13.3	.36	13.7	16.5	14.1	12.9	12.1	14.2	.58
Neuropathy	19.2	22.6	25.0	8.5	43.3	20.5	.001	13.4	19.0	12.8	10.1	17.5	14.2	.002
Nephropathy	31.2	38.7	47.5	25.0	40.0	25.3	.009	18.3	23.4	25.0	15.4	25.4	12.7	.002
Cerebrovascular	21.7	35.5	22.5	14.4	28.3	22.9	.006	17.6	24.2	14.1	14.4	23.3	17.5	.006
Cardiovascular	61.2	74.2	62.5	54.8	71.7	57.8	.03	43.9	49.6	46.8	38.0	53.8	44.3	.001
Peripheral vascular disease	18.5	32.3	17.5	9.0	36.7	16.9	.001	12.0	25.4	10.9	6.1	20.0	10.2	.001
Metabolic	3.5	1.6	5.0	3.7	3.3	3.6	.92	0.5	0.4	1.3	0.3	0.8	0.3	.43
DCSI														
							.001							.001
0	21.9	14.5	20.0	26.6	6.7	28.9		38.7	35.1	38.5	41.5	32.9	39.5	
1	13.6	11.3	10.0	17.0	15.0	8.4		13.7	6.9	12.8	16.0	14.2	13.6	
2	19.2	14.5	12.5	21.3	15.0	24.1		16.7	13.7	14.1	18.5	14.2	18.1	
3	12.5	14.5	10.0	12.8	16.7	8.4		9.7	12.1	9.0	10.4	6.3	9.0	
4	12.0	9.7	12.5	14.4	15.0	6.0		10.0	9.3	15.4	8.1	12.9	9.9	
5+	20.8	35.5	35.0	8.0	31.7	24.1		11.2	23.0	10.3	5.6	19.6	9.9	

^a^ Two-sided χ^2^ test of association between race and ethnicity and each diabetes-related complication and diabetes complications severity index (DCSI).

Diabetes and complications were positively associated with pancreatic cancer (Table 4). We observed that individuals with diabetes had 48% increased odds (odds ratio [OR] = 1.48, 95% confidence interval [CI] = 1.14 to 1.91) of pancreatic cancer compared with those without diabetes. Cardiovascular disease (OR = 1.88, 95% CI = 1.45 to 2.44), nephropathy (OR = 1.75, 95% CI = 1.32 to 2.33), and metabolic disease (6.61, 95% CI = 2.49 to 17.50) were positively associated with pancreatic cancer risk after adjusting for demographic, lifestyle characteristics, and chronic conditions, including diabetes.

**Table 4.** Relative risk of pancreatic cancer in relation to the presence of diabetes and diabetes-related complications compared with the absence of each condition

Chronic condition	No. of cases/No. of controls	OR^a^ (95% CI)		OR^b^ (95% CI)	
Diabetes (yes vs no)	197/524	1.46 (1.12 to 1.88)		1.48 (1.14 to 1.91)	
Diabetes-related complications					
Retinopathy (yes vs no)	71/236	0.82 (0.59 to 1.13)		0.85 (0.61 to 1.19)	
Neuropathy (yes vs no)	83/232	1.14 (0.84 to 1.55)		1.09 (0.79 to 1.49)	
Nephropathy (yes vs no)	135/316	1.72 (1.30 to 2.27)		1.75 (1.32 to 2.33)	
Cerebrovascular (yes vs no)	94/304	0.92 (0.69 to 1.24)		0.93 (0.68 to 1.27)	
Cardiovascular (yes vs no)	265/758	1.80 (1.40 to 2.30)		1.88 (1.45 to 2.44)	
Peripheral vascular disease (yes vs no)	80/209	1.08 (0.77 to 1.52)		1.11 (0.79 to 1.57)	
Metabolic (yes vs no)	15/8	5.84 (2.26 to 15.09)		6.61 (2.49 to 17.50)	

^a^ Conditional logistic regression model adjusted for education, neighborhood-level socioeconomic status (SES), alcohol intake, smoking status, any vigorous physical activity, body mass index (BMI), and matched factors (age, race, sex) were used to define strata. CI = confidence interval; OR = odds ratio.

^b^ Conditional logistic regression model adjusted for education, neighborhood-level SES, alcohol intake, smoking status, any vigorous physical activity, BMI, diabetes, and other comorbid conditions and matched factors (age, race, sex) were used to define strata.

Risk of pancreatic cancer increased with increasing DCSI score (Table 5). Compared with no complications, a greater risk of pancreatic cancer was found in relation to the DCSI as follows: 1 complication (OR = 1.61, 95% CI = 1.11 to 2.33), 2 complications (OR = 1.94, 95% CI = 1.38 to 2.74), 3 complications (OR = 2.26, 95% CI = 1.52 to 3.37), and 4 or more complications (OR = 2.59, 95% CI = 1.82 to 3.69). When we replaced DCSI categories with the count of complications as a linear variable, for every 1-unit increase in DCSI score, participants had 18% greater risk of pancreatic cancer (OR = 1.18, 95% CI = 1.11 to 1.25). We observed that the continuous measure of DCSI (for every 1-unit increase in score) was associated with a 22%-23% increased risk of pancreatic cancer among patients with (OR = 1.22, 95% CI = 1.09 to 1.36) and without diabetes (OR = 1.23, 95% CI = 1.11 to 1.36).

**Table 5.** Relative risk of pancreatic cancer and DCSI by diabetes status

Risk factor	No. of cases/No. of controls	OR^a^ (95% CI)		OR^b^ (95% CI)	
Total cohort (n = 2161)					
DCSI (continuous)	—	1.20 (1.14 to 1.26)		1.18 (1.11 to 1.25)	
DCSI (categorical)					
0	95/669	1.00 (Referent)		1.00 (Referent)	
1	59/236	1.77 (1.24 to 2.54)		1.61 (1.11 to 2.33)	
2	83/289	2.10 (1.51 to 2.93)		1.94 (1.38 to 2.74)	
3	54/167	2.41 (1.65 to 3.54)		2.26 (1.52 to 3.37)	
4+	142/367	3.02 (2.22 to 4.11)		2.59 (1.82 to 3.69)	
Without diabetes (n = 1422)					
DCSI (continuous)	—	1.18 (1.10 to 1.27)		1.23 (1.11 to 1.36)	
With diabetes (n = 739)					
DCSI (continuous)	—	1.12 (1.05 to 1.19)		1.22 (1.09 to 1.36)	

^a^ Conditional logistic regression adjusted for matched factors (age, race, sex) were used to define strata. CI = confidence interval; DCSI = diabetes complications severity index; OR = odds ratio.

^b^ Conditional logistic regression adjusted for education, neighborhood-level socioeconomic status, alcohol intake, smoking status, any vigorous physical activity, body mass index, diabetes, and other comorbid conditions and matched factors (age, race, sex) were used to define strata.

## Discussion

In our study, the number of complications from diabetes was associated with an elevated risk of pancreatic cancer even after controlling for a number of well-studied risk factors including obesity, smoking history, and other chronic conditions. Metabolic disease, cardiovascular disease, and nephropathy were also associated with increased risk of pancreatic cancer, independent of other risk factors and irrespective of diabetes status.

Several epidemiological studies have reported that diabetes is associated with an increased risk of pancreatic cancer (9,12). We know that diabetes diagnosed at a later age (12), those with incident diabetes (12), and those with diabetes for a longer duration (25) are at an increased risk of pancreatic cancer, but these studies measured only the presence or absence of disease. To the best of our knowledge, no studies have linked DCSI and pancreatic cancer risk. Previous epidemiological studies have reported that DCSI and complications are associated with an increased risk of mortality (14), more hospitalizations (14), and higher health-care use (15) and that Hispanics and African Americans have a higher prevalence of complications (15), but these studies do not link complications to risk of cancer. Our hypothesis was that complications are associated with increased risk of pancreatic cancer because they would indicate a higher DCSI and a higher probability of damage to the pancreas.

It remains unclear whether the association between diabetes and pancreatic cancer is due to hyperglycemia, whether diabetes is a marker of underlying biologic factors that alter cancer risk such as insulin resistance and hyperinsulinemia, or whether the association between cancer and diabetes is indirect and due to common risk factors such as obesity (26). Our study suggests that some complications such as nephropathy, cardiovascular disease, and metabolic conditions are independently associated with pancreatic cancer incidence. Among known pancreatic cancer risk factors, several medical conditions such as diabetes and those found in our study are associated with increased risk pointing to a chronic inflammatory hypothesis (27,28). The hypothesis suggests that chronic conditions and complications resulting from unmanaged disease can result in chronic inflammation, and the risk of pancreatic cancer should increase for those conditions that are related to inflammation. Gomez-Rubio and colleagues (29) used a systems approach to characterize high-risk patients based on multimorbidities and found that patients who had been diagnosed with 3 or more metabolic conditions, including diabetes, had 61% increased odds of pancreatic cancer (95% CI = 1.11 to 2.35) compared with no conditions. Whereas some complications were independently associated with pancreatic cancer, we also found a positive association between the frequency of complications and a patient’s pancreatic cancer risk.

The goal of this study was to examine the relationship between DCSI and pancreatic cancer risk and whether DCSI can be used to identify patients at high risk and has the utility of being implemented in clinical practice without surveying patient’s self-reported chronic conditions. We cannot rule out reverse causation in our study; however, our study adds to the evidence that diabetes and associated complications are associated with risk of pancreatic cancer. In a sensitivity analysis, the distribution of DCSI was similar across stage at diagnosis of pancreatic cancer (not shown); therefore, the observed positive association is likely not the result of detection bias and suggests that the DCSI score is not an indicator for underlying pancreatic cancer that is not yet diagnosed. Further, we examined the DCSI in a 2-year period and observed multiple complications; however, more longitudinal research is needed to determine if the associations are consistent with diabetes-related complications that occur well before cancer onset.

We found that African American and Latino participants were more likely to be diagnosed with 5 or more diabetes complications compared with other racial and ethnic groups, which is similar to other findings (15); however, we also add to the literature that understudied Native Hawaiians have a higher prevalence of complications compared with whites (30). Our results are particularly striking because minority patients are at an increased risk of diabetes and are more likely to have uncontrolled disease with a higher prevalence of complications. This suggests that they may be at higher risk of developing pancreatic cancer. The incidence of pancreatic cancer is indeed higher for African Americans (31,32) and Native Hawaiians (33,34). Although we did find that Latinos were more likely to have 5 or more complications, evidence suggests that they do not have a higher incidence of pancreatic cancer compared with other racial ethnic groups (12). More research is needed to understand whether accounting for complications explains the higher incidence of pancreatic cancer in racial and ethnic minority groups.

Our study has several strengths. Our study had a large proportion of racial and ethnic minorities, and we were able to account for important confounders such obesity (3,6,7) and smoking (3) by using linked lifestyle baseline questionnaire data to cancer registry data and Medicare claims. Furthermore, our study used medical claims data to identify diabetes complications, which can be used to identify high-risk patients and can be translated into clinical practice.

However, our study is not without limitations. First, in this study, we did not assess the pharmacological treatment of diabetes with metformin or insulin. Although the biological basis for these treatments is not fully understood, a meta-analysis of 37 studies has shown that metformin is associated with 46% reduced risk of pancreatic cancer (35). Metformin may reduce the risk of cancer because it can prevent worsening diabetes and related complications. In addition, long-acting insulin compared with no insulin may be associated with an increased risk of pancreatic cancer although it may just be an indicator of long-lasting and/or severe diabetes (36). The focus of our study, however, was to examine whether patients with complications are at an increased risk of cancer compared with those without diabetes or those with diabetes alone. Pharmacologically unmanaged disease likely results in greater complications from diabetes. Patients with a higher DCSI score may be less likely to take appropriately dosed medications than those without complications (37) and would therefore bias the observed results toward the null. Second, we did not account for the length or duration of diabetes because the goal of this study was to examine complications within 24 months of diagnosis. Given the start of Medicare coverage at aged 65 years only, we could not systematically ascertain the time of first diabetes for all cohort members. Finally, our study included a racially diverse group of people from a large prospective cohort study who were enrolled in Medicare FFS and older than aged 65 years. Because Medicare-eligible individuals covered by health maintenance organization plans, such as Kaiser, may have different risk factor and disease profiles, findings in this population may have been different, and although approximately two-thirds of pancreatic cancer cases are older than 65 years and the average age at the time of diagnosis is 70 years (1), our study results should not be generalized to people outside of this group.

In conclusion, our results in the MEC suggest that diabetes-related complications are associated with an elevated risk of pancreatic cancer compared with patients without complications. The results suggest the utility of the DCSI to assess risk of pancreatic cancer when medical claims are readily available, particularly for health plan administrators, provider practices, and researchers. Furthermore, more long-term longitudinal research is needed to rule out whether diabetes-related complications are capturing underlying pancreatic cancer and to examine whether the DCSI score can be used as a way to identify and potentially screen high-risk patients.

### Funding

This study was supported in part by the National Cancer Institute (grant no. R01CA209798, PI: VWS; and U01CA164973, PI: LLM), and a diversity research supplement (grant no. R01CA209798-02S1, PI: VWS).

### Notes

**Role of the funder:** The funders had no role in the design of the study; the collection, analysis, and interpretation of the data; the writing of the manuscript; and the decision to submit the manuscript for publication.

**Conflicts of interest:** The authors have no financial conflicts of interest to disclose.

**Acknowledgments:** We acknowledge the efforts of the National Cancer Institute. The interpretation and reporting of these data are the sole responsibilities of the authors.

**Presentation of results:** Results were presented at the Annual Meeting of the American Association of Cancer Research on November 7, 2019, in Maui, Hawaii.

**Author Contributions**: AJF, AHW, SJP, and VWS: Study concept and design. LLM, LRW, KRM, and GM: Acquisition of data. All coauthors: Analysis and interpretation of data. AJF, AHW, and VWS: Drafting of the manuscript. All coauthors: Critical revision of the manuscript for important intellectual content. AJF, JP, and LRW: Statistical analysis. AJF and VWS: Obtained funding. JP, LLM, LRW, and KRM: Administrative, technical, or material support. AJF: Study supervision.
